# Fuzzy Clustering of Maize Plant-Height Patterns Using Time Series of UAV Remote-Sensing Images and Variety Traits

**DOI:** 10.3389/fpls.2019.00926

**Published:** 2019-07-17

**Authors:** Liang Han, Guijun Yang, Huayang Dai, Hao Yang, Bo Xu, Haikuan Feng, Zhenhai Li, Xiaodong Yang

**Affiliations:** ^1^College of Architecture and Geomatics Engineering, Shanxi Datong University, Datong, China; ^2^Key Laboratory of Quantitative Remote Sensing in Agriculture of Ministry of Agriculture, Beijing Research Center for Information Technology in Agriculture, Beijing, China; ^3^National Engineering Research Center for Information Technology in Agriculture, Beijing, China; ^4^College of Geoscience and Surveying Engineering, China University of Mining and Technology-Beijing, Beijing, China

**Keywords:** FCM, temporal profile, maize, plant height, clustering

## Abstract

The application of high-throughput phenotyping (HTP) techniques based on unmanned aerial vehicle (UAV) remote-sensing platforms to study large-scale population breeding opens the way to more efficient acquisition of dynamic phenotypic traits and provides new tools that should help close the gap between genotyping and traditional field-phenotyping methods. Toward this end we used a field UAV-HTP platform to deploy a RGB high-resolution camera to acquire time-series images. By using three-dimensional reconstructed point cloud models, we developed a repeatable processing workflow to extract plant height from time-series images. The plant height determined by the UAV-HTP platform correlated strongly with that measured manually. The plant heights estimated at various growth stages form temporal profiles that give insights into changes and trends in genotyping. Based on fuzzy c-means clustering analysis, we extract the typical dynamic patterns in phenotypic traits (i.e., plant height, average rate of growth of plant height, and rate of contribution of plant height) hidden in the temporal profiles. The fuzzy c-means clustering and set-intersection operation were first applied to analyze the temporal profile to identify how plant-height patterns change and to detect differences in phenotypic variability among the genotypes. The results revealed the capacity of UAV remote sensing to easily evaluate field traits on multiple timescales, for a few breeding plots or for 1000s of breeding plots.

## Introduction

Maize (*Zea mays* L.) is one of the most important grain crops in China. According to a report by the National Bureau of Statistics in China, the planting area and grain yield of maize in 2017 were 35.45 million hectares and 21.58 million tons (National Bureau of Statistics of China, 2017), respectively, ranking it first among the major crops. China’s maize imports are expected to increase gradually to 7.2 million tons by 2024 and 2025 (United States Department of Agriculture (USDA), 2015). Genetic breeding has contributed to increasing maize yield and to ensuring global food security. New technologies to accelerate breeding through improving genotyping and phenotyping methods are currently in demand (Tester and Langridge, 2010).

An accelerated breeding pipeline to obtain breeding-target-related agronomic traits is a key to developing improved varieties (Shakoor et al., 2017). High-throughput phenotyping (HTP) techniques based on unmanned aerial vehicles (UAV-HTPs) in field breeding programs have gradually become promising tools with which to acquire phenotype traits with high temporal and spatial resolution, affordable cost, and non-invasive remote-sensing methods (Araus and Cairns, 2014). UAV-HTP can identify and access both simple and complex phenotypic traits, which are the key breeding targets for genetic breeding and include grain yield (Kefauver et al., 2017; Herrmann et al., 2019), above-ground biomass (Han et al., 2019), lodging resistance (Han et al., 2018a), senescence (Makanza et al., 2018), and plant height (Pugh et al., 2018; Wang et al., 2019).

Plant height in maize is an important agronomic trait because it is highly heritable (Peiffer et al., 2014), easy to measure, and influences the stalk lodging (Li et al., 2007). Previous research has shown that plant height correlates highly with biomass or grain yield, so it is used for estimating biomass (Salas Fernandez et al., 2009; Han et al., 2019) and grain yield (Yin et al., 2011; Barrero Farfan et al., 2013; Geipel et al., 2014). Manually measuring plant height in the field is usually only done at the end of growth. However, the expression of each quantitative trait locus (QTL) controlling plant height depends on the time at which the measurements are made and on the space where they are made (Yan et al., 2003; Wang et al., 2015). Pauli et al. (2016) found that the correlation between HTP canopy traits, including plant height, and agronomic traits varies over time. Measuring plant height throughout crop growth can provide new insights to genetic breeding, but it is time consuming (Chang et al., 2017).

Previous studies have shown that the use of HTP technologies to monitor multi-temporal crop height and growth has various advantages (Holman et al., 2016; Duan et al., 2017; Kronenberg et al., 2017; Malambo et al., 2018; Thompson et al., 2019). For example, Liebisch et al. (2015) used a Zeppelin airship as a remote-sensing platform to acquire multi-sensor and multi-temporal images throughout the maize growth season and found that the traits of various genotypes differ clearly. However, they gave no detailed agronomic interpretation. Pugh et al. (2018) used unmanned aerial systems to determine plant height in maize and sorghum and formed high-resolution temporal growth curves that provided new physiological insights and applications for phenotyping. Many clustering algorithms have been adopted in the literature to extract expression patterns from time-series data, such as density-based clustering for analyzing the electrical load profile (Yang et al., 2018), hierarchical clustering for genetic diversity (Tagliotti et al., 2018), and fuzzy clustering for gene expression (Olsen et al., 2006; Collins et al., 2013; Piening et al., 2018).

In the present study, we used a UAV-based high-throughput platform to collect RGB images in a field breeding program and a method to extract plant-height information from the images. The plant height acquired at different growth stages and from different genotypes can be combined to form temporal profiles, which offer novel insights into the diversity of gene expression. The specific objectives for this study included (i) developing a repeatable processing workflow to extract plant height from time-series images, (ii) investigating the accuracy of plant-height estimates by comparing them with field measurements, and (iii) detect differences in phenotypic variability among the genotypes.

## Materials and Methods

### Field Experiments

**FIGURE 1:**
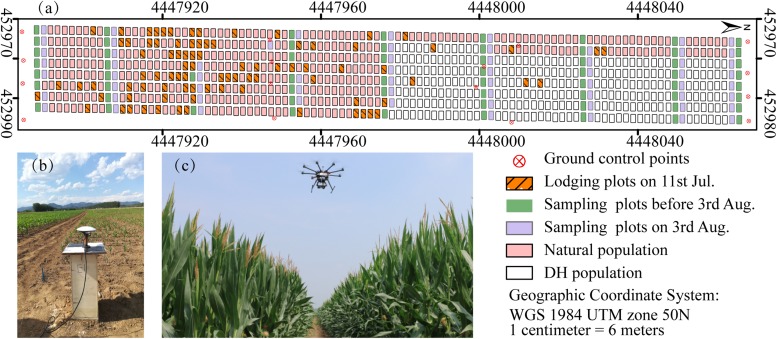
Maize breeding experiment at Xiao Tangshan National Precision Agriculture Research Center, Changping, 2017. **(a)** Study area. **(b)** Ground control points (GCPs) measurement using a differential global positioning system. **(c)** UAV platform.

The maize-breeding experiments were conducted in 2017 at the research station of Xiao Tangshan National Precision Agriculture Research Center of China, Changping District of Beijing City (115°50′ 17′′–116°29′ 49′′ E, 40°20′ 18′′–40°23′ 13′′ N). The experimental field was approximately 27 m wide and 210 m long, comprising 800 breeding plots in total, with each plot being 2 m (3 rows) wide and 2.4 m long (Figure 1a). The single factor experiment design was applied to explore the differential expression of maize genotypes. Eight hundred breeding plots were divided into four sub-populations according to the genetic background: mixed, TEM (temperate) and TST (tropical and subtropical) and DH (doubled-haploid). The first three sub-populations, i.e., 482 breeding plots, were used to search for patterns in the temporal profiles of the plant height. Since the ground truth data included the DH subpopulation, its samples were also used as the validation dataset. The experiment used a solid row and column configuration with a row spacing of 0.6 m and a column spacing of 0.8 m. Eight hundred plots were planted on May 15, 2017 at a seeding density of 6 plants m^–2^.

Prior to the first flight, 16 ground control point (GCP) markers consisting of 45 cm × 45 cm black and white square planks were evenly distributed in the experimental field, and the XYZ coordinates of each GCP marker were measured by using a differential global positioning system (DGPS, South Surveying & Mapping Instrument, Co., Ltd., Guangzhou, China) with millimeter accuracy (Figure 1b). According to the row number from south to north, about every 10 rows set up a group of sampling plots, for a total of nine groups with eight plots in each group. The sampling plots in campaigns 2–4 were the same (see Table 1). Some sampling plots (19.4%, i.e., 56:288) were excluded due to abnormal growth or lodging, so the sample size of ground truth varied at different observation date. Three plants were selected at random in the center of the sampling plots to measure plant height at four time points. The manual measurement of plant height was done by using a telescopic leveling rod. The mean height of the three plants was used as the canopy height of the given sampling plot for ground truth.

**TABLE 1:** Timing of measurement campaign.

**Campaign**	**AGL (m)**	**Time points**	**Plant height**	Growth stage^*^
1	40	2017-06-08 (24)	−	V4
2	60	2017-06-29 (45)	2017-06-29 (45)	V10
3	60	2017-07-11 (57)	2017-07-11 (57)	V14
4	60	2017-07-28 (74)	2017-07-29 (75)	VT
5	60	2017-08-04 (81)	2017-08-03 (80)	R1

The date and days after sowing (DAS) are given for each campaign. AGL, above ground level. ^*^The leaf collar method of Ritchie et al. (1993) was used for staging maize plant growth.

### UAV Campaigns and Image Processing

A UAV (DJI Spreading Wings S1000, SZ DJI Technology, Co., Shenzhen, China) equipped with a RGB high-resolution camera (DSC-QX100, 5472 × 3648 pixels, Sony Electronics, Inc., Tokyo, Japan) was used to capture the RGB images after sowing at five time points (Figure 1c). ISO and shutter speed were fixed at 160 and 1/2000, respectively. Flight paths over the experimental area were determined by using the DJI PC ground station (SZ DJI Technology, Co., Shenzhen, China) to ensure substantial overlap (i.e., 80% forward and 75% side) with two different flight altitudes above ground level (AGL),i.e., 40 and 60 m, and a flight speed of 6 m/s, yielding six strips. To classify the ground point cloud and build the digital elevation model (DEM), the flight altitude AGL for the first flight on June 8, 2017 was 40 m, yielding a ground-sampling distance of 0.72 cm. The corresponding image-acquisition dates and maize growth stage are given in Table 1.

**FIGURE 2:**
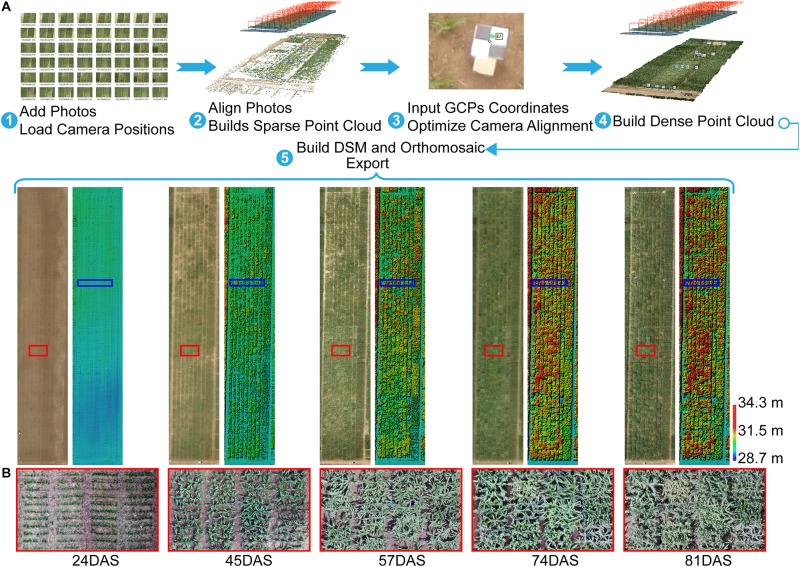
Agisoft Photoscan processing workflow and export for orthomosaic and DSM. **(A)** Five-step semi-automated processing workflow. **(B)** Orthomosaics with zoomed views of area (red rectangle) show differences in maize growth at the plot scale. For example, some plots were lodged at 57DAS, and some plots had tassels at 74DAS, whereas others did not. GCP, ground control point; DSM, digital surface model; DAS, days after sowing.

After acquiring images by using the UAV with a RGB camera at multiple different time points, the images were processed by using Agisoft PhotoScan (version 1.3, Agisoft LLC, Saint Petersburg, Russia) to generate orthomosaics and digital surface models (DSMs) with the GCPs. The GCPs were used to optimize the camera position and orientation data, which led to better results for generation Agisoft (2018). A semi-automated processing workflow was applied to export a short time series (five time points) of orthomosaics and DSMs according to the days after sowing (DAS) (Figure 2A).

### Point Cloud Classification and Plant-Height Extraction

#### CSM and DEM Generation

Crop surface models (CSMs), which are widely used to obtain plant-height information from various crops, were introduced by Bendig et al. (2014) for barley, Han et al. (2018a) for maize, De Souza et al. (2017) for sugarcane, and Holman et al. (2016) for wheat. They can be obtained by subtracting the DEM from the DSMs. As mentioned above, the DSM can be generated directly and then exported by using the Agisoft Photoscan software.

**FIGURE 3:**
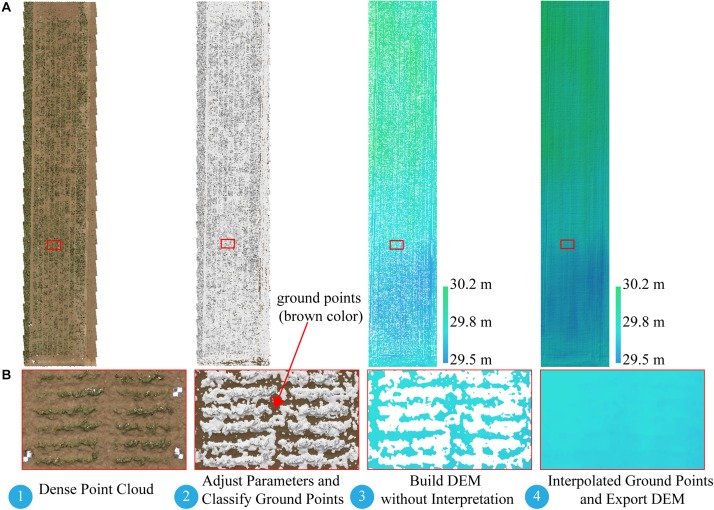
Workflow for building DEM based on point cloud classification. **(A)** Full extent of dense point cloud and DEM. **(B)** Expanded views of area (red rectangle). DEM, digital elevation model.

The difficulty of this study is how to build a DEM. Several studies extracted soil point elevations from a DSM that was not covered by vegetation and built a DEM by using Kriging spatial interpolation (Yue et al., 2017; Han et al., 2018a) or inverse-distance-weighted interpolation (Brocks and Bareth, 2018). To ensure an accurate DEM, we manually picked up a large number of soil points when using the interpolation method to build a DEM, which was time consuming and offered a low degree of automation. With the help of the Agisoft Photoscan software, we introduced an alternative approach to build a DEM with a triangulation-based ground classifier. The goal was to divide early low-vegetation-cover dense point clouds (on June 8, 2017) into ground point clouds and the rest and build the DEM based only on classified ground point clouds. The adaptive triangulated irregular network ground classifier in the Agisoft Photoscan software is an iterative algorithm that works basically as follows (Serifoglu Yilmaz et al., 2018): (1) breaking dense point clouds into cells of a certain size (cell size) and detecting the lowest points of each cell, (2) triangulating these points to obtain an approximate ground model, and (3) adding new points to the ground class, providing that it satisfies two conditions: (i) limiting its distance from the ground model to a given maximum distance, and (ii) keeping the angle between the ground model and the line connecting this new point with a point from ground class within a certain maximum angle. This third step is repeated until all points are checked. These parameters (cell size, maximum distance, and maximum angle) are adjusted until we get an acceptable point cloud classification. Blanks left after the exclusion of non-ground points can be filled with nearest neighbor point interpolation. For campaign 1, we used a trial-and-error method to find an acceptable point cloud classification result with a cell size of 20 cm, a maximum angle of 1.5°, and a maximum distance of 3 cm, and then built the DEM based only on the classified ground point clouds. Figure 3 illustrates the workflow for building a DEM-based point cloud classification.

#### Plant Height Estimation

After building the CSMs, they were processed via ArcMap software (version 10.2, Esri, Inc., Redlands, CA, United States) and ENVI software (version 4.5, Esri, Inc., Redlands, CA, United States) to extract plant-height information. Up to this point, the CSM has been a raster image mixed with soil background and plant-height information for the various vertical levels. Using the mean to calculate plant height at the plot level may result in underestimation, especially in areas where vegetation coverage is low. To solve this problem, we used an image-segmentation method based on vegetation index (i.e., normalized green-red difference index) to segment plants from bare soil. NGRDI values for soils are always recorded as negative (Shimada et al., 2012). The *NGRDI* was calculated by using

(1) $$N\,G\,R\,D\,I=\frac{g\,r\,e\,e\,n-r\,e\,d}{g\,r\,e\,e\,n+r\,e\,d}$$

where *green* and *red* are the reflection in the green band and red band of the remote-sensing images, respectively.

**FIGURE 4:**
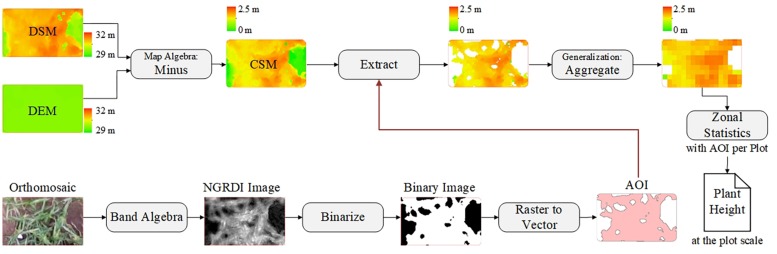
Workflow for plant-height extraction at plot level. AOI, areas of interest.

The orthomosaic image was operated on in both bands to obtain the NGRDI image, and then NGRDI image was binarized to separate vegetation and non-vegetation areas by using ENVI software. The vegetation areas were converted into a vector map as areas of interest, which served as a mask to extract the plant-height information from the CSM, yielding a new CSM based only on images of vegetation. The pixels in the new CSM were aggregated to filter out low-level plant-height information. The Zonal statistics tool in ArcMap software was used to calculate the mean of the above results per plot by using the areas of interest. Figure 4 shows the corresponding workflow for plant-height extraction at the plot level.

Where Green and Red are the reflection in the green band and red band of the remote-sensing images, respectively.

### Temporal Profiles Phenotypic Traits

The plant-height dataset was constructed by using the plant-height extraction method described in Section “Point Cloud Classification and Plant-Height Extraction” and the time series of UAV orthomosaic, which provided five time-point profiles. The temporal profile revealed that dynamic changes in plant height at different development stages were regarded as a phenotypic trait in our study. Breeders and agronomists are interested not only in the changes in plant height, but also in the distribution of plant-height increments during the different development stages. Therefore, two other temporal profile traits were derived (Han et al., 2018b): the average growth rate of plant height (AGRPH) and the contribution rate of plant height (CRPH). The following equations were used to calculate AGRPH and CRPH, respectively:

(2) $$\begin{array}{c} A\,G\,R\,P\,H_{P_{k}\,T_{i}}=\frac{P\,H_{P_{k}\,T_{i+1}}-P\,H_{P_{k}\,T_{i}}}{T_{i+1}-T_{i}} \end{array}$$

(3) $$C\,R\,P\,H_{P_{k}\,T_{i}}=\frac{P\,H_{P_{k}\,T_{i+1}}-P\,H_{P_{k}\,T_{i}}}{P\,H_{P_{k}\,T_{4}}}\times 100\%$$

where subscripts *P*_*k*_ and *T*_*i*_ represent plot *k* and time point *i*, respectively. AGRPH is the ratio of plant-height increment to day increment between two adjacent time points and represents the increment per day. CRPH is the percent contribution of plant-height increment to the final plant height and reflects the incremental distribution at different development stages.

### Clustering of Temporal Profiles

During data preparation before clustering, the raw dataset with the three traits were cleaned by deleting abnormal records that stemmed from abnormal growth or lodging on July 11. Outliers were treated by using the capping-flooring approach. Outliers were capped at a certain value above the 98th percentile value or floored at a certain value below the 2nd percentile (Pyle, 1999).

We are interested in whether genotypes can express phenotypic traits in similar patterns, so the temporal profiles were clustered by using the fuzzy c-means (FCM) clustering algorithm. This study uses the R package ‘e1071’ (version 1.7-0) to implement this algorithm (David et al., 2018), which is based on the open-source statistical language R (R Core Team, 2018^1^). Fuzzy c-means is a data-clustering algorithm in which each profile belongs to more than one cluster with varying degrees of membership in the range [0, 1]. The centroid of a cluster is the mean of all points weighted by their degree of belonging to the cluster (Kesemen et al., 2016). It uses Euclidean distance as the distance metric. With the FCM algorithm, the difficulty lies in choosing suitable values for the parameters *C*, which defines the optimal number of clusters, and *M*, which defines how fuzzy the cluster is. The greater *M* is, the fuzzier the cluster will be in the end. Pal and Bezdek (1995) obtained the optimal range of m from the experimental study of clustering validity as [1.5, 2.5], and considered that the median *M* = 2 was acceptable in general. Bezdek (1993) found that *M* = 2 had the clearest physical meaning. The FCM parameter was therefore set to *M* = 2 for the following analysis. The parameter *C* was chosen by computing six indices, and the best number of clusters is determined by using the majority rule (Charrad et al., 2014). The majority rule is an unweighted voting rule with a threshold of 50%. The six indices used were the partition coefficient (PC), the partition entropy coefficient (PE), the Xie-Beni index (XB), the Fukuyama-Sugeno index (FS), the fuzzy hyper volume (FHV), and the partition density index (PD). Table 2 describes these indices in detail.

**TABLE 2:** Six indices for determining cluster size.

**Indices**	**Temporal profiles**	**Proposed cluster size**	**Criteria**
Fuzzy hyper volume (FHV)	PH	2	Minimum value of the index. Small FHV indicates presence of compact clusters based on concepts of hyper volume and density (Gath and Geva, 1989).
	CRPH	2	
	AGRPH	2	
Partition density (PD)	PH	2	Maximum value of the index. PD is the general partition density. According to the physical definition of density; larger value of PD indicates better clustering (Gath and Geva, 1989).
	CRPH	2	
	AGRPH	2	
Xie-Beni (XB)	PH	2	Minimum value of the index. XB measures the average intra-cluster fuzzy compactness against the minimum inter-cluster separation. The optimal cluster size is reached when the minimum of XB is found (Xie and Beni, 1991).
	CRPH	2	
	AGRPH	2	
Fukuyama-Sugeno (FS)	PH	2	Minimum value of the index. Small FS indicates compact and well-separated clusters (Zanaty, 2012).
	CRPH	2	
	AGRPH	2	
Partition coefficient (PC)	PH	2	Maximum value of the index. The closer the index is to 1.0, the crisper the clustering. When PC is close to 1/*C*, no clustering trend exists in the data (Zanaty, 2012).
	CRPH	2	
	AGRPH	2	
Partition entropy (PE)	PH	2	Minimum value of the index. Ranges over the interval [0, log*C*]. When a PE is close to upper bound, and no clustering trend exits in the data (Krawczyk and Cyganek, 2017).
	CRPH	2	
	AGRPH	4	

*PH, plant height; AGRPH; the average growth rate of plant height; CRPH, the contribution rate of plant height.*

Based on FCM clustering analysis, typical dynamic patterns of phenotypic traits (i.e., PH, CRPH, or AGRPH) that are hidden in the temporal profiles were extracted and are represented by the plots of the cluster centroids. The R package ‘UpSetR’ (version 1.3.3) visualized the dataset intersections (Conway et al., 2017). The intersections of clusters and genetic backgrounds were used to identify and explain typical dynamic patterns. When a genetic background dominates a cluster, the centroid profile of the cluster was chosen as the typical dynamic pattern of a phenotypic trait with this genetic background. The following two inequalities were used to identify the dominant genetic background.

(1) More than one third of the samples in a cluster from a given genetic background, which is called the *ClusterProportion*.

$$C\,l\,u\,s\,t\,e\,r\,\mathrm{Pr}o\,p\,o\,r\,t\,i\,o\,n=\frac{N\,G_{i\,n\,c\,l\,u\,s\,t\,e\,r}}{N\,C_{t\,o\,t\,a\,l}}\geq \frac{1}{3}$$

Where *NG*_*incluster*_ is the sample size in the cluster from a given genetic background, *NC*_*total*_ is the sample size of the cluster.

(2) Samples from a given genetic background in a cluster accounted for more than 2/3 of the total sample from this genetic background, which is called the *TotalProportion*.

$$T\,o\,t\,a\,l\,\mathrm{Pr}o\,p\,o\,r\,t\,i\,o\,n=\frac{N\,G_{i\,n\,c\,l\,u\,s\,t\,e\,r}}{N\,C_{t\,o\,t\,a\,l}}\geq \frac{2}{3}$$

where *NG*_*total*_ is the total sample size from a given genetic background.

## Results

### Reconstruct Digital Surface Model and Orthomosaic

To evaluate the accuracy of geolocation of DSMs and orthomosaics, Table 3 summarizes the root mean squared errors (RMSEs) of GCPs (in cm) and the two performance indices for restructuring the DSM. The point cloud density can impact the quality or accuracy of the DSM, which is based on point clouds. The smaller the resolution value is, the higher is the resolution of DSM and the more accurate the DSM is depicted. The total GCP error, calculated by using the ground truth of GCPs (measured by using a DGPS; see section “Field Experiments”) and their reconstructured locations in the UAV images, varied over campaigns from 1.45 to 6.56 cm and were considered reasonable and acceptable, taking into account the flight altitude and allowing for error (<10 cm) (Roth and Streit, 2017; Malambo et al., 2018). The lower the AGL is, the smaller are the total errors, the higher is the DSM resolution, and the larger is the point density. In the latter two campaigns, the GPS marks were more easily occluded by leaves and pollen, resulting in poor geolocation accuracy (i.e., the total error is 6.56 and 6.10 cm, respectively). Because the errors in both the horizontal and vertical direction increased significantly, it is reasonable to suspect that GPS markers were inadvertently and slightly moved during the field activities. Errors in horizontal direction strongly affected the geolocation of automatic areas of interest extracted from orthomosaics (see section “Point Cloud Classification and Plant-Height Extraction”) and further affected the accuracy of plant-height extraction.

**TABLE 3:** Summary of geolocation accuracies and performance of reconstructed DSM.

**Campaign**	**X error (cm)**	**Y error (cm)**	**Z error (cm)**	**Total error (cm)**	**Resolution (cm/pix)**	**Point density (points/cm^2^)**
1	0.94	0.98	0.48	1.45	1.44	47.9
2	1.15	1.39	0.64	1.92	2.65	14.2
3	1.28	1.46	0.73	2.08	2.71	13.7
4	5.44	2.08	2.34	6.56	2.46	16.5
5	4.90	1.56	3.29	6.10	2.23	20.2

*The XYZ error is in a certain direction and the total error is the root mean square error (RMSE) of the GCP markers. The RMSE is used to evaluate the geolocation accuracy of orthomosaics and DSMs. The resolution and point density serve to evaluate the performance of the reconstructed DSM.*

### Plant-Height Estimation and Validation

**FIGURE 5:**
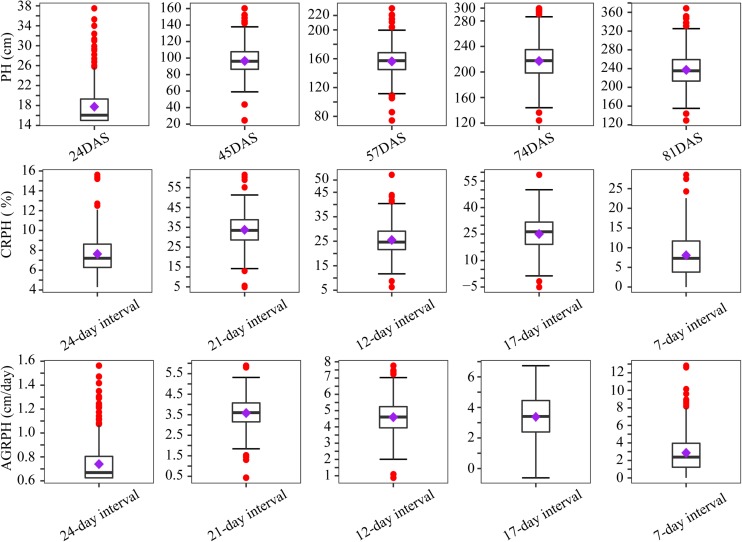
Boxplots showing distribution of phenotypic traits before treating outliers. Boxplots are based on three traits from 400 plots. The solid line in the box indicates the median and the purple square is the mean. The red points are outliers. PH, plant height; PH, plant height; AGRPH, average growth rate of plant height; CRPH, contribution rate of plant height; DAS, days after sowing.

By using the method in Section “Point Cloud Classification and Plant-Height Extraction,” the plant height of 400 plots containing a natural population was extracted from the five-time-point series orthomosaic. Figure 5 shows the distribution of three phenotypic traits before treating outliers. Outliers occurred most frequently in the first campaign, because the plant height was low at that time (the mean plant height was less than 20 cm) and plants were sparse, which is not conducive to UAV remote-sensing observation due to apparent noise artifacts actually caused by sparse plant representation. Most of the outliers were removed after applying the capping-flooring treatment.

**FIGURE 6:**
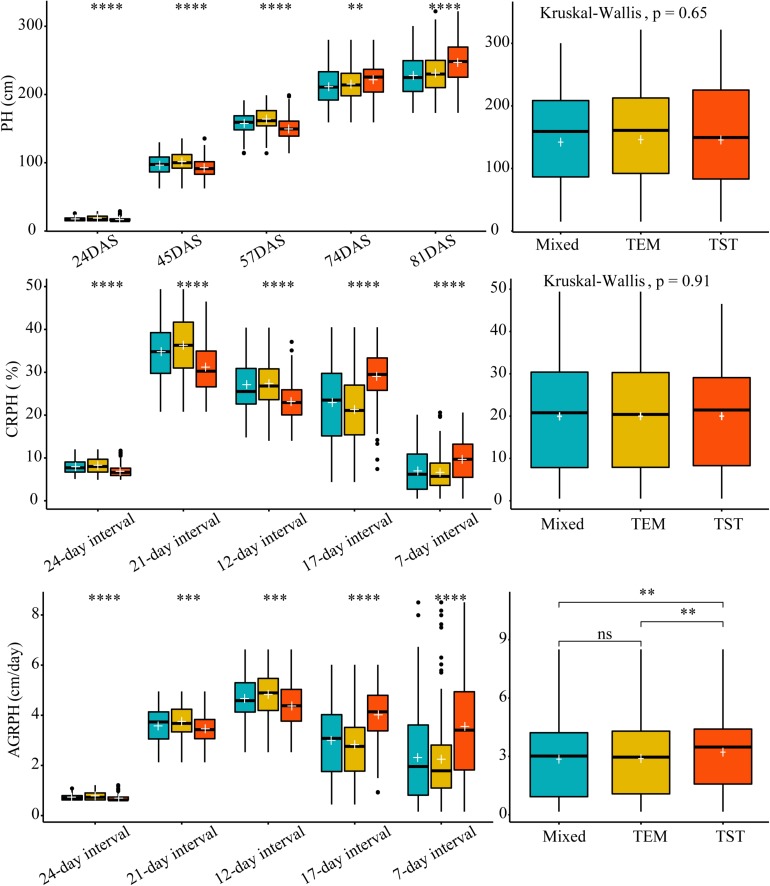
The difference in three traits based on different genetic backgrounds. Kruskal–Wallis test was used to determine whether there was a significant difference in each trait among the three genetic backgrounds (i.e., mixed, TEM, and TST). The white plus sign indicates the mean. The following convention for symbols indicates statistical significance: *p* > 0.05 (ns); ^*^p ≤ 0.05; ^∗∗^*p* ≤ 0.01; ^∗∗∗^*p* ≤ 0.001; ^∗∗∗∗^*p* ≤ 0.0001. PH, plant height; AGRPH, average growth rate of plant height; CRPH, contribution rate of plant height; DAS, days after sowing. TEM, temperate; TST, tropical and subtropical.

We used Kruskal–Wallis test to determine whether there was a significant difference in each trait among the three genetic backgrounds. Kruskal–Wallis test found no significant differences in overall PH and CRPH among the three genetic backgrounds (*p* > 0.05, Figure 6 from right side). In terms of a specific growth stage, there were significant differences in each trait among the three genetic backgrounds (Figure 6 from left side). The most obvious finding to emerge from the test was that observing traits from time dimension was easy to find differences in phenotypic traits among different genetic backgrounds.

**FIGURE 7:**
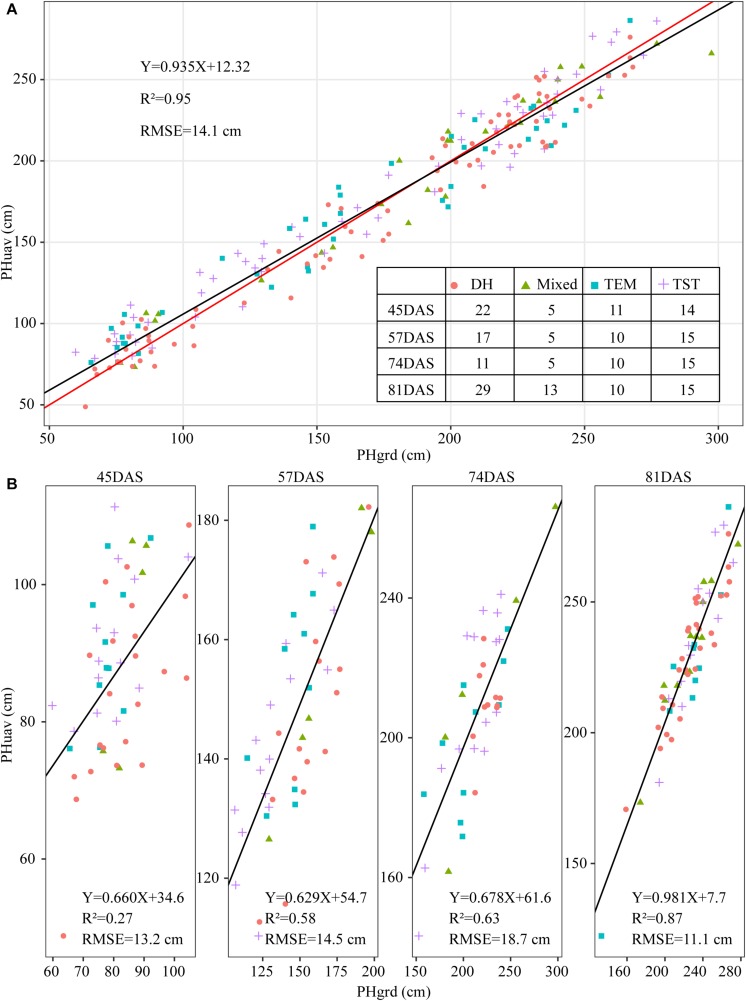
Scatter plot of plant height extracted from UAV images (PHuav) versus manual ground measurements made with rulers (PHgrd). **(A)** Along the growing season. **(B)** Individual observation time point. The blue solid line represents the regression line and the red solid line has unity slope. The cross tabulation at the upper-right corner shows the frequency distribution of sampled plots according to four genetic backgrounds and four time points. DAS, days after sowing; TEM, temperate; TST, tropical and subtropical.

To validate the accuracy of the plant-height estimates, the mean height extracted from UAV images of the sampled plots (i.e., PHuav) was compared with the mean height as determined by manual measurements with rulers (i.e., PHgrd). Figure 7A compares PHuav with PHgrd and shows that the two have a strong and statistically significant (*p*-value < 2.0 × 10^–16^) linear relationship, with *R*^2^ = 0.95 (RMSE = 14.1 cm). The UAV measurements underestimate the plant height significantly in campaigns 4 (74DAS) and 5 (81DAS), which may be because PHgrd was measured at the top of the tassels after tasseling. However, reconstructing the point cloud of the tassels from UAV images at an AGL of 60 m is difficult because of their small spindles and complex branches. Figure 7B shows that, unlike regression statistics along the growing season, for the individual observation time point, the linear relationship between manual and UAV based heights is weak. In terms of R-squared (R^2^, the coefficient of determination), there is an increasing trend toward the linear relationship from campaigns 2 (45DAS) to 5 (81DAS), with the gradual closure of the canopy.

Because of differences in genotypes, maize in different plots may be in different development stages. For example, the TEM population was in the flowering stage while the TST population was still in the vegetative stage (Figure 2B). This heterogeneity in the development of maize may also lead to a high RMSE (18.7 cm in Figure 7B). Due to the tall stature of terminal maize growth, last season height measurements may be biased or less accurate due to difficulties in taking consistent measurement above eye level. Any analysis of the correlation between UAV measurements and manual measurements may be challenging because it assumes that the manual measurements are correct and that the UAV measurements must therefore reproduce them (Pugh et al., 2018). Therefore, due to the subjectivity of observers, the high RMSE may reflect the subjectivity of the manual observers and shows that three repeated manual measurements may not be sufficient in this study.

### Determination of Cluster Size

**FIGURE 8:**
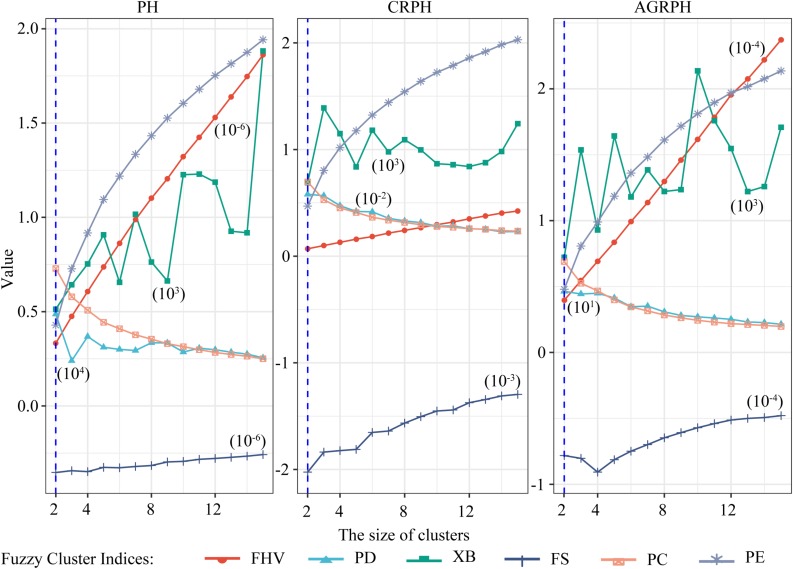
Determining optimal size of clusters based on six fuzzy-clustering indices. The blue vertical dashed line indicates the optimal size of clusters for PH, CRPH, and AGRPH. To ensure that six indices can be presented simultaneously in the same coordinate system, some indices are scaled (i.e., PE, XB, PD, and FHV). The figures in brackets are scaling coefficients. PH, plant height; AGRPH, average growth rate of plant height; CRPH, contribution rate of plant height; FHV, fuzzy hyper volume; PD, partition density; XB, Xie-Beni; FS, Fukuyama-Sugeno; PC, partition coefficient; PE, partition entropy.

The size of clusters *c* was varied between 2 and 15. We iterated 500 times to ensure convergence and explored the combination of clustering size and fuzzy parameter *M* = 2 and found the optimal partition with *C* = 2 and *M* = 2 based on the majority rule. For clustering temporal profiles of PH and CRPH traits, we obtained a consistent optimal cluster size from the six fuzzy clustering indices (i.e., *C* = 2), but for clustering temporal profiles of AGRPH, five fuzzy clustering indices proposed *C* = 2, but PE proposed *C* = 4 (Figure 8 and Table 2).

### Detecting Typical Temporal Profiles

**FIGURE 9:**
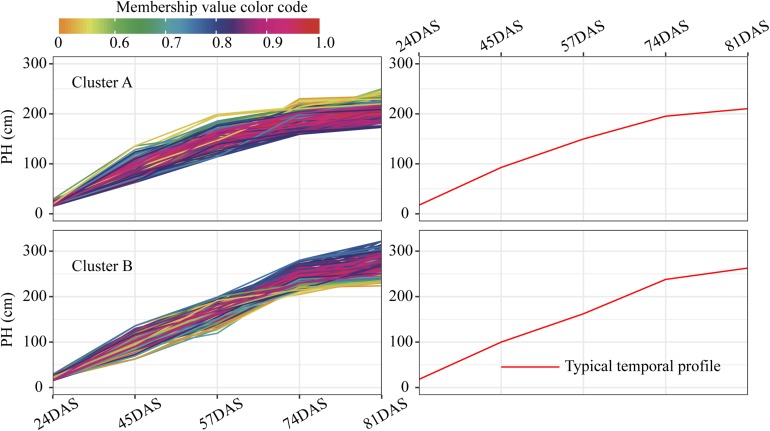
Clustering temporal profiles of PH. Temporal profiles are assigned to clusters A and B by fuzzy c-means clustering. Each trace is color coded according to its membership in the respective cluster (see color bar). The right plot shows a polyline formed joining the clustering centroids at five time points that is used to identify the dynamic pattern of the PH trait. PH, plant height; DAS, days after sowing.

For each trait, 400 temporal profiles from three genetic backgrounds fell into different clusters after FCM clustering, and each profile was assigned a membership grade for the clusters. To better understand the dynamic pattern of each trait, we join the clustering centroids at five time points by a polyline to form a typical temporal profile. Data visualization analyses reveal a number of typical patterns. For the profiles of PH observed in clusters A and B (Figure 9), the upward trends are similar, except for the large differences in plant height between the 74DAS (mean = 195.4 versus 237.8 cm) and 81DAS (mean = 210.4 versus 262.8 cm). Although the TST population accounted for 48.5% of cluster B, the total proportion was only about 58% (100:172), so we conclude that no dominant genetic background exists in cluster B, and no further explanation is needed (Figure 12A). These results suggested that the typical temporal profile of PH was not conducive to detecting plant height variations among different genotypes of maize.

**FIGURE 10:**
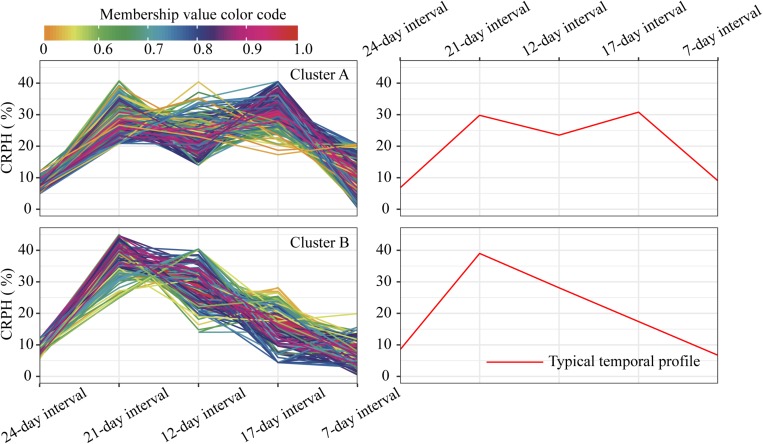
Clustering temporal profiles of CRPH. Temporal profiles are assigned to clusters A and B by fuzzy c-means clustering. Each trace is color coded according to its membership in the respective cluster (see color bar). The right plot shows a polyline formed joining the clustering centroids at five time points that is used to identify the dynamic pattern of the CRPH trait. CRPH, contribution rate of plant height.

For the profiles of CRPH observed in clusters A and B (Figure 10), the TST population accounts for 60.5% in cluster A, and the total proportion is as high as 80.2% (138:172), so we conclude that the TST population forms the dominant genetic background of cluster A. The typical temporal profile of cluster A is thus used to represent the dynamic pattern of the CRPH traits in the TST population (Figure 12B). At the second, third, and fourth time points, the CRPH of the TST population remains above 25%, especially at the fourth time point (17-day interval), where the CRPH increases to over 30%. This indicates that the TST population was in the vegetative stage from the second to the fourth time points. Because the plant height will reach its maximum when a plant enters into the reproductive stage (at or shortly after growth stage VT) from the vegetative stage (Mcwilliams et al., 1999). When planted in the northern temperate regions, the effective accumulated temperature for the TST population is insufficient, so it takes a longer to enter the reproductive growth stage from the vegetative growth stage. In other words, the growth cycle is usually prolonged. The consequence is that the accumulated temperature for reproductive growth is insufficient to produce high grain yields (Hatfield and Prueger, 2015). These results suggested that the typical temporal profile of CRPH could detect the difference of plant height increment among different genotypes of maize.

**FIGURE 11:**
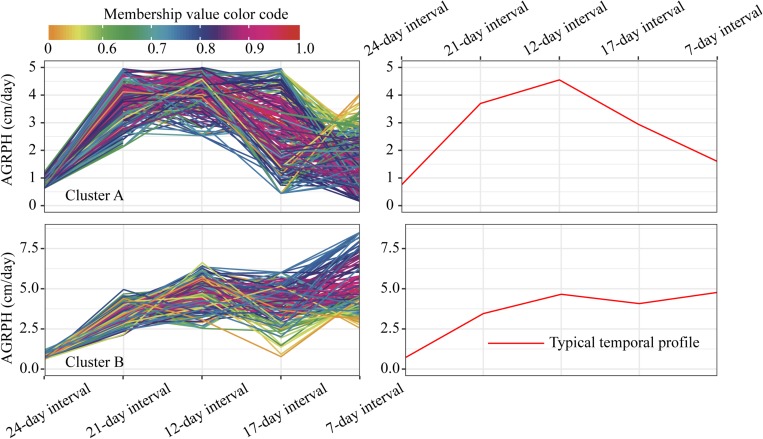
Clustering temporal profiles of AGRPH. Temporal profiles are assigned to clusters A and B by fuzzy c-means clustering. Each trace is color coded according to its membership in the respective cluster (see color bar). The right plot shows a polyline formed joining the clustering centroids at five time points that is used to identify the dynamic pattern of the AGRPH trait. AGRPH, average growth rate of plant height.

**FIGURE 12:**
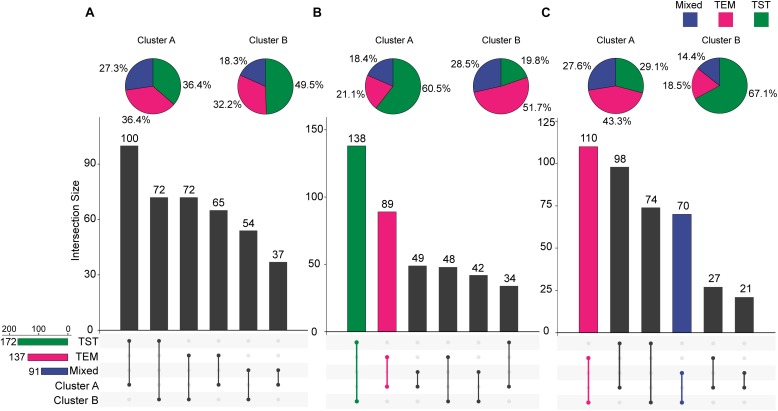
Intersections between clusters and genetic backgrounds. **(A)** PH trait. **(B)** CRPH trait. **(C)** AGRPH trait. Pie chart shows the population of the three genetic backgrounds (i.e., mixed, TEM, and TST) in a cluster. Colored dumbbell and column represent the intersection and the dominant genetic background in a cluster, respectively. PH, plant height; AGRPH, average growth rate of plant height; CRPH, contribution rate of plant height; TEM, temperate; TST, tropical and subtropical.

For the profiles of AGRPH observed in clusters A and B (Figure 11), TEM population accounts for 43.3% in cluster A and 80.2% (110:137) in total (Figure 12C). Therefore, we conclude that the TEM population is the dominant genetic background of cluster A, and the typical temporal profile of the cluster may be used to explain the dynamic pattern of the AGRPH trait in the TEM population. The phenomenon whereby the growth rate observed in the TEM population first increases and then decreases from the vegetative growth stage to reproductive growth stage. Although the total percent of the mixed population in cluster A is 76.9% (70:91), we cannot reasonably explain the dynamic pattern of the AGRPH trait due to the unclear source of the genetic background. These results suggested that the typical temporal profile of AGRPH could detect the difference of plant height growth rate among different genotypes of maize.

## Discussion

### Factors Affecting Accuracy of Maize Plant-Height Extraction

Although it achieved higher accuracy and lower estimation error, the accuracy with which the maize plant height is extracted can still suffer from uncertain factors. First, misclassification may cause some classified dense points to not represent the real ground when creating a DEM, resulting in an underestimate of plant height (Geipel et al., 2014). Unfortunately, the GCPs markers were not placed in time before seedling emergence after sowing. Although the vegetation coverage was low, the plants were small, and the soil could be exposed over large areas on June 8th, 2017, which could minimize the possibility of misclassification, it is clear that collecting remote-sensing images and creating a DEM before emergence can completely avoid the problem of misclassification. When planting in a heterogeneous field or canopy closure, less soil is exposed and this method is more prone to misclassification.

Second, GCP distribution and stability factors have a certain impact on the accuracy with which plant height is extracted. Tonkin and Midgley (2016) report that the distribution and quantity of GCPs strongly influence the quality of a model’s reconstruction (e.g., DEM and DSM). To facilitate an accurate reconstruction, GCPs should be located at the edge or outside of the study area, and the quantity of GCPs should be sufficient (James and Robson, 2012). Han et al. (2018a) found uneven topographic changes in the southern part of the study area, which should be considered to increase the number of GCPs appropriately in this part. To collect UAV remote sensing time-series images, it is recommended to periodically check whether the GCP markers have moved. If so, they should be accurately restored their original position before making the UAV flight.

**FIGURE 13:**
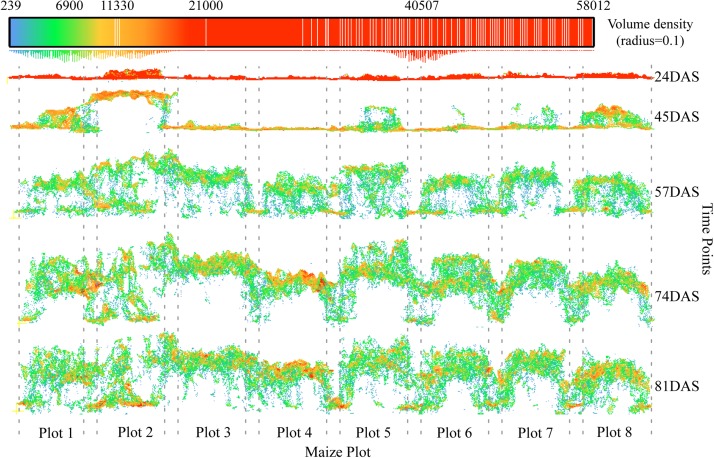
Characteristics of canopy development and changes in point cloud density. The positions of the eight plots in the example are given in Figure 2 (blue rectangles). The position of the high-density point cloud changes as the maize canopy develops. The dotted line represents the boundary of a plot.

Third, the characteristics of the development of the maize canopy structure could introduce errors to varying degrees during the different growth stages. From a horizontal perspective, Figure 13 shows that high-density point cloud locations do not always appear at the top of the canopy, but shift continuously as the canopy develops. At the second time points (45DAS), the number two maize plot shows an optimal canopy structure for extracting plant height; that is, high-density point clouds have all gathered at the top of the canopy to form a horizontal structure like a balance beam. However, in most cases, depending on the canopy structure, high-density point clouds may appear at any vertical position, which is the most essential cause of underestimation.

The best way to avoid this problem is to remove the low-level point cloud in the vertical direction while maintaining the high-level canopy spatial structure, so as to ensure that multiple plants in a plot can participate in the plant-height calculation. Han et al. (2019) has presented a method for extracting plant height that takes into consideration the maize canopy structure. The core steps of this method include the spatial Kriging interpolation based on multiple neighboring maximum pixels from multiple plants. In comparison, we adopt a simpler aggregation analysis method whereby we aggregate pixels within a certain window size by using the maximum value, and then calculate the mean of these local maxima, which serves as the representative plant height at the plot scale. The biggest difference between the two approaches is that our method does not use the spatial Kriging interpolation. Compared with the percentile height method (Li et al., 2015; Kronenberg et al., 2017; Malambo et al., 2018), the distinct advantage of extracting plant height by considering the canopy spatial structure is to ensure that multiple plants in a plot can participate in the calculation, which theoretically reduces the errors caused by the high-density point cloud at low levels (see, e.g., Plot 2 at 81DAS in Figure 13) or caused by outliers when the growth of maize plants are uneven at the plot scale.

### Clustering of Temporal Profiles

To search for patterns in the temporal profiles of these traits (i.e., PH, CRPH, and AGRPH), we explored several clustering methods and found that FCM as a soft clustering method is more suitable for our analysis than is a hard clustering method such as k-means or hierarchical clustering. The initial points of the k-means clustering algorithm are randomly selected, which can cause unstable clustering results. Bubeck et al. (2012) showed that the initialization strongly influences the k-means clustering results. Hierarchical clustering does not require us to pre-specify the number of clusters to be produced, but once the clusters are merged or divided, it cannot be corrected and the quality of the clustering is limited (Oded and Lior, 2010).

One major shortcoming of these hard-clustering methods is that they make an either-or decision regarding the temporal profile clustering (Kim et al., 2006). Many genotypes may have the same temporal profile. FCM clustering provides more information regarding the degree of membership of each temporal profile to each cluster of genotypes. The main advantage of the FCM is its ability to handle noisy data (Halkidi et al., 2001). The shortcomings of the FCM are that the clustering result is sensitive to *M* and the best value of *M* depends on the dataset used. Therefore, the value of *M* should be interpreted with caution.

Distance measures quantify the dissimilarity between the two clusters. In this study, we use the Euclidean distance. Han et al. (2018b) used a shape-based distance metric to cluster these traits and obtained more-typical temporal profiles (called “typical curves” in their article). However, due to the excessive number of typical temporal profiles, the agronomic interpretation of the clustering solutions is not clear. Based on our research, typical temporal profiles can better identify genetic differences at different stages of crop development.

Note that temporal resolution affects the interpretation of temporal profiles. Changing temporal resolution may lead to changes in dynamic trait patterns. It is impractical to measure these traits at high frequencies during the crop growth cycle, and may even have a negative impact on breeding (Araus and Cairns, 2014). Therefore, careful consideration and understanding of the appropriate time points for phenotyping field traits is critical for their evaluation (Shakoor et al., 2017). In view of this, our future work will focus on determining the best remote-sensing observation time scale to identify stable and reliable dynamic patterns of traits, according to the crop growth cycle.

## Conclusion

This study identifies dynamic patterns of maize plant height from a short time series of UAV orthomosaic in a field breeding program. First, by using the reconstructed three-dimensional point cloud model based on RGB images and a new method for extracting plant height, we estimate plant heights from different genotypes at five time points, thereby forming multi-temporal profiles that provide insights into the changes and trends in plant height. Second, based on FCM clustering analysis, typical dynamic patterns of three phenotypic traits (i.e., PH, CRPH, and AGRPH) hidden in temporal profiles were extracted and represented by plotting the cluster centroids. Based on our research, typical temporal profiles regarded as traits could allow better identification of genetic differences at different stages of crop development. Typical temporal profiles could enable new ways to understand phenotypic traits, as demonstrated herein by the three highly detailed traits reflecting plant height. This concept can be extended from traits involving temporal plant height to other traits, such as spectral vegetation index, canopy coverage, or biomass.

Although the capacity of UAV remote sensing to scale phenotyping up from a few to 1000s of breeding plots allows breeders to effortlessly assess the development of field traits on multiple time scales and thereby accelerate the breeding of novel traits, limitations remain that must be considered. For example, some sensors are expensive, and data processing takes a long time. In particular, another urgent issue is whether the phenotypic features obtained by remote sensing by UAV can be accurately marked by QTL analysis.

## Data Availability

All datasets for this study are included in the manuscript and the supplementary files.

## Author Contributions

LH drafted and revised the manuscript. GY proposed the conceptualization of this study and reviewed the manuscript. HD edited the manuscript. HY and LH conducted field experiments. BX and HF collected image data. LH, ZL, and XY analyzed and interpreted the results. All authors read and approved the final manuscript.

## Conflict of Interest Statement

The authors declare that the research was conducted in the absence of any commercial or financial relationships that could be construed as a potential conflict of interest.

## Footnotes

1 https://www.R-project.org/
